# Snacking Patterns in Children: A Comparison between Australia, China, Mexico, and the US

**DOI:** 10.3390/nu10020198

**Published:** 2018-02-11

**Authors:** Dantong Wang, Klazine van der Horst, Emma F. Jacquier, Myriam C. Afeiche, Alison L. Eldridge

**Affiliations:** Nestlé Research Center, Route du Jorat 57, Vers-chez-les-Blanc, 1000 Lausanne, Switzerland; Klazine.VanderHorst@rdls.nestle.com (K.v.d.H.); Emma.Jacquier@rd.nestle.com (E.F.J.); Myriam.AfeicheZehil@rdls.nestle.com (M.C.A.); Alison.Eldridge@rdls.nestle.com (A.L.E.)

**Keywords:** children, snacking, patterns, energy, nutrients

## Abstract

Snacking is common in children and influenced by many factors. The aim of this study is to provide insight of both common and country-specific characteristics of snacking among 4–13 year old children. We analyzed snacking prevalence, energy and nutrient contributions from snacking across diverse cultures and regions, represented by Australia, China, Mexico, and the US using data from respective national surveys. We found that the highest prevalence of snacking was in Australia and the US (over 95%) where snacking provided one-third and one-quarter of total energy intake (TEI), respectively, followed by Mexico (76%, provided 15% TEI) and China (65%, provided 10% TEI). Compared to 4–8 year-olds, the consumption of fruits and milk was lower in 9–13 year-old children, with a trend of increasing savory snacks consumption in China, Mexico, and the US. The nutrient density index of added sugars and saturated fat was higher, especially in Australia, Mexico, and the US. Results suggested that snacking could be an occasion to promote fruit and vegetable consumption in all countries, especially for older children. Snacking guidelines should focus on reducing consumption of snacks high in saturated fat and added sugars for Australia, Mexico, and the US, whereas improving dairy consumption is important in China.

## 1. Introduction

Snacking, also described as eating in-between meals, is prevalent among children in many parts of the world. Prevalence, frequency, energy and nutrient contributions from snacking vary from the west to the east, and from developed countries to developing countries, which could be represented by Australia, China, Mexico, and the United States (US). In the US, the prevalence of snacking among 2–18 year-olds already exceeded 90% in the 1990s [1], and it has reached 95% in 2013–2014 (2–19 year-olds) [2]. American children snack 2–3 times per day and snacking contributes 24% of their daily energy intakes [2]. A similar scenario was reported in Australia [3], with about 96% of children and adolescents reported consuming snacks in the 2011–2012 national survey, representing 30.5% of total daily energy intake. In contrast, the situation in China is quite different. The prevalence of snacking among 7–12 year-old children in China increased from 14% in 1991 to 54% in 2009, but the average snacking frequency remained at about once per day, and the energy contribution of snacking was still relatively low, i.e., 6–8% [4]. The snacking habits in Mexican children are intermediate, with 68% of 6–13 year-old children reporting snacking in 2012 with an average frequency of 1.2 times per day. Snacks contributed 19% of Mexican children’s daily energy intake [5].

There are many factors that influence food choices during snacking occasions, such as dietary culture, socio-economic status, as well as child age [6]. Foods and beverages consumed as snacks can improve the total diet [7], but due to the global epidemic of childhood obesity, there is also a valid concern about the excess amount of energy snacking may bring to the total diet. Increased snacking frequency has been positively associated with energy consumption [5,8] and with a higher odds of overweight and abdominal obesity in children [9]. Thus the nutritional quality of the foods and beverages consumed in-between traditional main meals and their potential impact on public health should be better understood.

Cross-country studies can provide useful insights and elucidate the diversity of snacking behaviors in children. This may help international organizations and nutrition policy makers to better understand the heterogeneity of this eating occasion. Dietary recommendations for snacking lack consistency and differ from country to country. In some countries, recommendations for snacking in children have been established [10,11], whereas in other countries, the dietary recommendations focus on the total diet and do not specifically address snacking [12,13,14]. Because snacking can contribute up to a third of daily energy in some countries, guidance is needed for both parents and children on appropriate food and beverage choices to ensure that snacking adds nutritional value without exceeding desirable energy intakes.

Our objective is to compare snacking behaviors, energy and nutrient contributions from snacks, and foods consumed during snacking across diverse cultures, regions, and socio-economic levels, represented by Australia, China, Mexico, and the US. To better understand the snacking habits of children in these countries, we conducted secondary analysis using data from existing national surveys. The aim of this paper is to explore the common and country-specific characteristics of snacking in terms of prevalence, foods and beverages consumed, and energy and nutrient contributions among children in these countries, and to suggest potential common and country-specific areas for improvement.

## 2. Materials and Methods

### 2.1. Study Population

This study used cross-sectional data from four national surveys: the Australian National Nutrition and Physical Activity Survey (NNPAS) [15], the China Health and Nutrition Survey (CHNS) [16], the Mexican National Health and Nutrition Survey (ENSANUT; Encuesta Nacional de Salud y Nutrición) [17], and the US National Health and Nutrition Examination Survey (NHANES) [18,19]. Secondary analysis was conducted using data from a total of 8586 4–13 year children, including 1576 from Australia, 1460 from China, 3864 from Mexico, and 1686 from the US. Children were grouped as 4–8 years-old (*n* = 4561) and 9–13 years-old (*n* = 4025) to reflect differences in nutritional needs and food consumption patterns, and also to be close to age-bands used for national nutrition recommendations.

The 2011–2012 Australia NNPAS is a nationally representative survey. Participants were selected using multi-stage area sampling in all Australian states and territories, covering both urban and rural households [15]. The CHNS is an ongoing longitudinal survey, but data for this study included only the 2011 survey year to be comparable with data from the other national surveys in our analysis. Participants were selected from nine provinces and three mega-cities (Beijing, Shanghai, and Chongqing) representing different geographies and economic development levels in China, using a multi-stage random cluster sampling method covering 228 communities in both urban and rural areas [16]. The 2012 ENSANUT is a cross-sectional multi-stage, stratified cluster-sample of 50,528 households representative of the Mexican population [17]. The 2013–2014 NHANES was collected from a multi-stage, stratified area probability sample of non-institutionalized individuals [18,19]. Data were weighted in Australia, Mexico, and the US according to the weights provided by Australia Bureau of Statistics, Encuesta Nacional de Salud y Nutrición, and the US National Health and Nutrition Examination Survey, respectively, but unweighted in China due to the lack of available population weighting information.

### 2.2. Dietary Assessment

All of these surveys collected 24 h dietary recalls using trained interviewers. Parents or caregivers completed the interviews or assisted with interviews for younger children according to the age limits defined by each country. In the NNPAS, ENSANUT, and NHANES, the automated multiple-pass methodology was used to collect the 24 h recalls [20,21]. In China, dietary intake data for CHNS were collected with 24 h recalls on three consecutive days combined with weighted estimates of household consumption of oils, sugar, salt, and condiments. Food models, picture aids, and household measures were used in all surveys to assist in portion size estimations. Second day 24 h recalls were collected on a sub-group of participants in Australia, Mexico, and the US to estimate usual nutrient intakes, but since our focus was on snacking in the context of daily intake, we used only day one of each individual’s 24 h recall. The first day of the three-day recall was used in China.

National food composition databases were used to estimate energy and nutrient intakes in each country. The Australian Food Composition Database [22], developed by Food Standards Australia and New Zealand was used for NNPAS. In China, energy and nutrient intakes were calculated based on the 2002 Chinese Food Composition Tables [23]. To estimate the intakes of some important missing nutrients, such as added sugar, the Chinese food codes were linked to USDA food codes [24] and the missing nutrient intakes were imputed using values from the US Department of Agriculture (USDA) Food and Nutrient Database for Dietary Studies (FNDDS) 2013–2014 [25]. In Mexico, national food composition tables were used for approximately 67% of the foods [26], and data from the 2011–2012 USDA National Nutrient Database for Standard Reference, release 26 [27] was used for the remaining 33% of foods missing from the national tables. Energy and nutrient intakes in NHANES were estimated from the USDA’s Food and Nutrient Database for Dietary Studies (FNDDS) 2013–2014 [25].

In each national survey, foods and beverages were assigned to food groups according to systems developed for each country. Although similar structure is used in all systems, the food groups differ from country to country, the number of the top level of food groups vary from 24 in Australia [28], 20 in China [23], 11 in Mexico [29], to 14 in the US [30]. For the purpose of our cross-country comparison, food groups from each country were aligned with the USDA What We Eat in America (WWEIA) food grouping system [31]. The classification system incorporates approximately 150 mutually exclusive categories, arranged in hierarchies. We evaluated foods and beverages consumed as snacks at the second hierarchical level, e.g., milk, cheese, yogurt rather than Milk and Dairy.

### 2.3. Definition of Snacking

In all the surveys, participants were asked to designate the name of each eating occasion, but the names used for different eating occasions differed between countries. We defined the main meals of the day as follows: breakfast, brunch, lunch, dinner (Australia); breakfast, lunch, dinner (China); desayuno, almuerzo, comida, cena (Mexico and Spanish-speakers in the US); breakfast, brunch, lunch, dinner or supper (US). To standardize the definition of snacking in this study, we considered snacking to be any eating occasion outside of the main meals of the day. These could have been designated as snack, extended consumption, drink/beverage, morning tea, afternoon tea, supper (Australia only), or in Spanish: merienda, entre comida, botana, bocadillo, or tentempie. Snack consumers are those who reported having at least one snacking occasion according to the described definition on the day of the 24 h recall.

### 2.4. Statistical Analyses

Snacking prevalence, frequency, per capita energy and nutrient intakes, and the top foods consumed as snacks are presented for each country. A nutrient density index was calculated to examine the micronutrient contribution from snacks relative to their energy contribution. The nutrient density index is the ratio between the percentage of daily intake of a nutrient contributed from snacking to the percentage of daily energy contributed from snacking [32]. A nutrient density index higher than 1 indicates that the intake of that nutrient from snacks is relatively greater than the energy contribution.

A consistent analysis approach was used to ensure the comparability of data in different countries. Statistical analyses were performed using SAS (version 9.3, SAS Institute Inc., Cary, NC, USA) and Stata (Release 13.0, Stata Corp., College Station, TX, USA). Differences between the countries and age groups were tested using Chi-square (χ^2^) for prevalence data and Student’s *t*-test for means. Bonferroni adjustments were applied for multiple comparisons.

## 3. Results

Characteristics of the study populations and their snacking habits are presented in Table 1. The prevalence of snacking varied between countries with more than 95% of children in Australia and the US reporting snack consumption, and about 60–80% reporting snacks in China and Mexico. Younger children showed a slightly higher prevalence of snacking compared to their older counterparts in the same country. The percentage of total daily energy intake (TEI) from snacks was highest in Australia with 33–34% of energy coming from snack occasions. Snacking contributed 24–27% of daily energy intake in the US. About 15% of daily energy intake was from snacking occasions among Mexican children. The lowest energy contribution from snacks was found in China with only 8–12% of daily intake from snacking.

**Figure 1:**
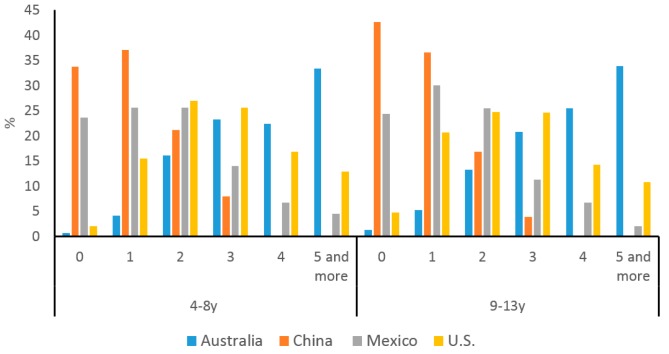
The distribution of snacking frequency among children from Australia, China, Mexico, and the US (%). Color of the bars represents different countries, blue for Australia, brown for China, grey for Mexico, and orange for the US. Notations of 4–8y and 9–13y refer to children 4–8 years of age and 9–13 years of age respectively.

The distribution of snacking frequency is shown in Figure 1. In both age groups, more than 30% children reported five or more snacking occasions per day in Australia, whereas in China over 70% of children either had no snack or only one snack in a day. In Mexico and the US, most children snacked 2–3 times a day.

The nutrient intakes during snacking occasions are shown in Table 2. Intakes varied from country to country due to the different choices of foods and beverages consumed during snacking occasions, the percent of children consuming each food category and the amount of consumption at each snacking occasion.

**Figure 2:**
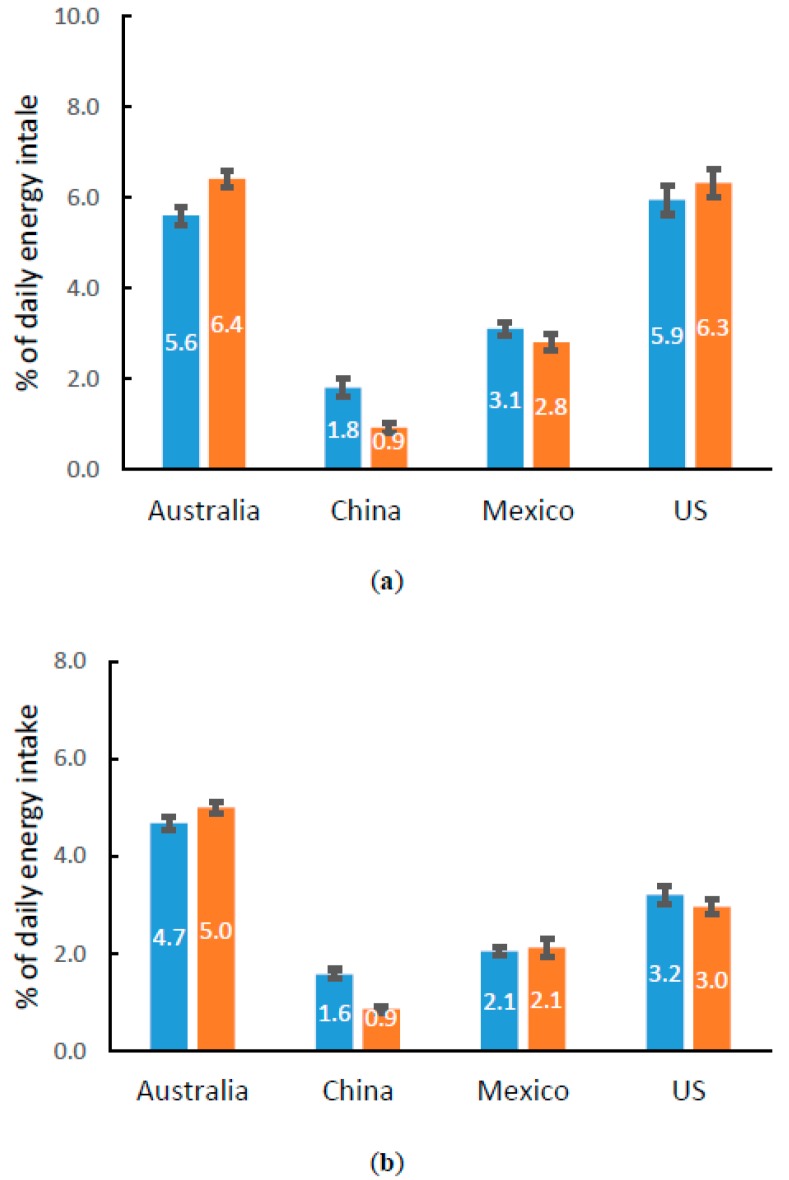
Energy contribution of added sugars and saturated fat during snacking occasions for children in Australia, China, Mexico, and the US (%). Data presented as Mean ± SE. Panel (**a**) and (**b**) show the percentages of energy contribution of added sugar and saturated fat, respectively. Numbers in the middle of bars are the percentages. Color of the bars represents age groups, blue for 4–8 years-old and brown for 9–13 years-old.

Added sugars and saturated fat are nutrients of potential concern during snacking, so we compared the energy contribution of added sugars and saturated fat from snacks (Figure 2). In the total daily diet, the recommendation for added sugar intake is consumption below 10% TEI [14,33]; for saturated fat it is below 10% TEI in the US [14] and below 8% in China [33]. The energy contribution of added sugars at snacking occasions was around 6% TEI in both age groups in Australia and the US, approximately 60% of the daily recommendation. The lowest values were found in China where added sugars contributed less than 2% TEI. Similarly, the saturated fat energy contribution was also high in Australia (4.7–5.0% TEI) and the US (3.0–3.2% TEI), and the lowest values were found in China (0.9–1.6% TEI).

**Figure 3:**
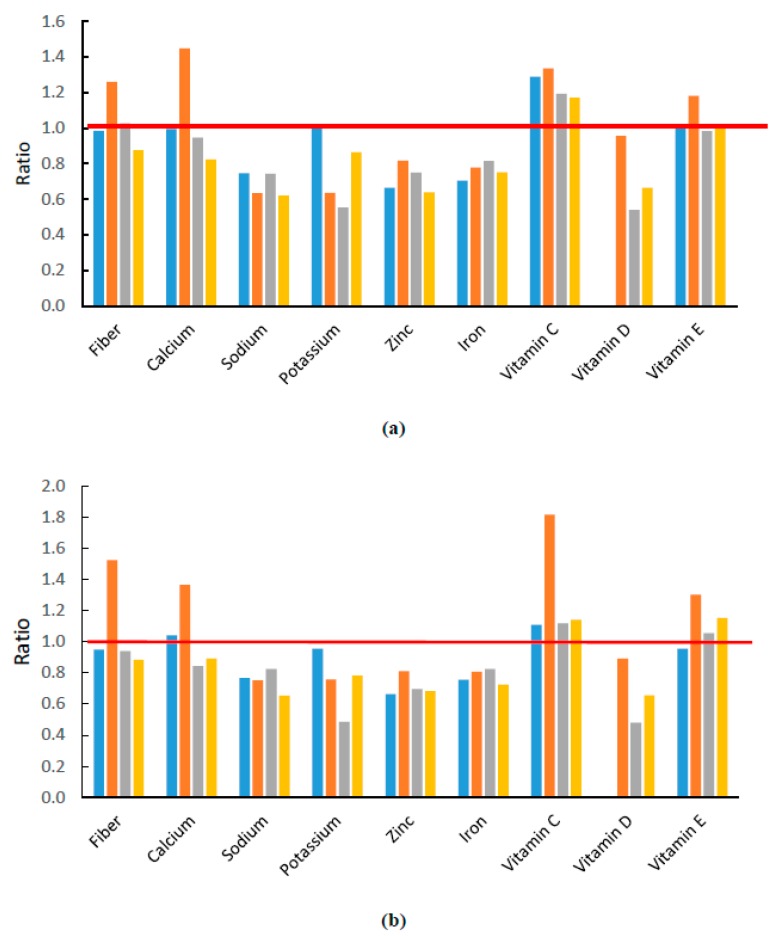
Nutrient density index of dietary fiber and micronutrient at snacking occasion as proportion. Panels (**a**) and (**b**) are the nutrient density index of 4–8 year-olds and 9–13 year-olds, respectively. The red line refers to a nutrient density index equal to 1. A nutrient density index greater than 1 indicates that the intake of that nutrient at snacking occasions is relatively higher than the energy contribution. Color of the bars represents different countries, blue for Australia, brown for China, grey for Mexico, and orange for the US.

To examine dietary fiber and micronutrient intakes at snacking occasions, the nutrient density index of each nutrient was calculated (Figure 3). We found that snacking contributed proportionally more vitamin C in all studied countries relative to its energy contribution. In China, snacking also provided disproportionally more dietary fiber, calcium, and vitamin E. On the other hand, the intakes of minerals, such as potassium, zinc, iron, and vitamin D were low relative to the energy contribution.

Foods and beverages consumed in-between meals varied from country to country, and from younger to older children (Table 3). A common feature is that fruits were an important component of snacking in all countries, especially among children 4–8 year. However, the importance declined for older children in all countries indicated by lower percentages of consumption. Other food groups, often more unhealthy ones, entered the top three, such as savory snacks in Mexico and the US.

In Australia, “water”, “fruits”, and “cookies & brownies” were the top three foods/beverages consumed in both younger and older children. The main energy-providing foods were “fruits”, “cookies & brownies”, and “savory snacks” in younger children, and “fruits”, “cookies & brownies”, and “milk” in the older ones. Compared to younger children, the percentages consuming “sweetened beverages” in older children was higher while “fruits” was lower. Despite no significant differences in the percentages of consuming, “candy” and “milk” contributed more energy in older children.

The most popular snacking foods and beverages in Chinese children were “fruits”, “crackers”, “milk”, and “yogurt” in both age groups. “Fruits”, “milk”, and “crackers” also provided the most energy during snacks. The percent consuming milk decreased from 16% in younger children to 8.4% in older children. “Meats” and “savory snacks” entered the top 10 in older children along with decreased consumption of “crackers” and “plant-based protein foods” consumption.

In Mexico, “water”, “fruits”, and “sweetened beverages” were the top three reported foods and beverages among younger children, whereas in older children, “savory snacks” replaced “fruits” and occupied the second place. “Fruits”, “savory snacks”, and “sweetened beverages” were the major energy contributors for all ages. The percent consuming of “savory snacks” increased from 18.5% in younger children to 24.2% in older children, along with a 90% increase in energy contribution. On the other hand, the percent of children having fruits decreased from 24.3% in younger children to 19.5% in older children.

In the US, the top three foods and beverages consumed were “water”, “candy”, and “fruits” in younger children, and “water”, “savory snacks”, and “candy” in older children. A significant reduction of the percent consuming “fruits”, “milk”, “cookies & brownies”, and “crackers” was found in comparing younger to older children. “Cheese” became one of the top 10 foods in older children.

## 4. Discussion

Foods and beverages consumed during snacking occasions are different from country to country, and from younger to older children. A mixed picture was revealed by this study, showing some healthy habits along with some worrisome issues. We found that the percentage of snack consumers, snacking frequency, and percentage of energy contribution from snacks were higher in Australia and US, followed by Mexico, and were lower in China. In Australia and US, the percentages consuming the top 10 food and beverage categories were higher than that of Mexico and China, likely reflecting that children in Australia and the US consumed a wider variety of foods and beverages as snacks.

A common feature of the foods and beverages reported during snacking was that “water” and “fruits” consistently appeared as two of the top three most popular choices, except in older US children, which reflects healthy habits. Water was not on the list in China since water intake information was not recorded in the survey, but other sources of information indicate that water is the most commonly consumed beverage among Chinese children [34]. Although “water” was the most reported item in snacking in Mexico, like in Australia and the US, the percent consuming was only 37% on a given day, which was much lower than that of Australia (83%) and US (65%). “Fruits”, another popular food consumed in this eating occasion, ranked among the top three in all countries in younger children, but dropped among older children. Similar trends were observed in “milk” consumption. Compared to younger children, the percent consuming “milk” was lower in older children, a trend also reported in a 2015 cross-country comparison of beverage consumption [34]. On the other hand, the consumption of “savory snacks” among older children increased in China, Mexico, and the US, both as percent consuming and the amount consumed. Thus, a common guidance for older children could be to reduce consumption of salty snack foods and replace them with “fruits”, “milk”, and “dairy foods” provided their total daily intakes do not exceed the recommendations. The promotion of healthy hydration is also needed, especially in Mexico [35].

The basis of dietary intake recommendations at the nutrient level is the relationship between people’s habitual dietary intake and its health implications. As part of the total diet, the nutritional value of snacking needs to be considered in a holistic manner. A common characteristic of total dietary intake across studied countries was the high intake of sugar and sodium. For instance, the US recommendation for added sugars intake is less than 10% TEI [14]. A recent study reported that added sugars contributed about 16% to TEI in 4–13 year children in the US, and almost 90% the children exceeded this recommendation [29]. Similarly, added sugars accounted for 12–13% TEI in Australian [36] and Mexican children [37]. If comparing to World Health Organization (WHO) free sugars intake recommendation where all monosaccharides and disaccharides added to foods are included, the situation would be worse. High daily sodium intake is another common issue among children in the studied countries. More than 60% of Chinese children had an excess sodium intake [24]. An Australian study measured daily sodium excretion and showed an equivalent salt intake level at 6 g/day [38]. The mean sodium intake in US school age children was over 3 g/day [39], much higher than the WHO recommendation (2 g/day sodium or 5 g salt) [40] and Dietary Guidelines for Americans recommendation (2.3 g/day) [14]. Although the sodium intake did not stand out disproportionally to its energy contribution in this report, salt-dense foods, such as savory snacks consumed at snacks, would contribute to daily sodium intakes. The trend of increasing ‘savory snacks’ consumption in older children described above deserves public attention, and should be addressed in nutrition education programs.

Sugars and sodium reduction is important in preventing non-communicable diseases. One of the key components of the success in UK salt reduction program started in 2003/2004 is the collaborative work between the food industry and the government to reduce population salt intake via gradual food reformulation [41]. The WHO also proposes that food product reformulation as an essential element of an overall approach to providing healthier food supply and preventing and controlling non-communicable diseases [42]. Thus, the involvement of food manufacturers is needed to improve the nutrition profile of commercially available snack foods. This approach should be combined with nutrition education and intervention programs to help to change children’s food choices and improve their nutrient intakes [43]. Parents and caregivers need to be part of the solution. Effective intervention strategies target the family as a whole, not just children, and engage parents directly [44]. However, the perception of parents and caregivers is that snack foods consist of candy, cookies, etc., rather than fruits, vegetables, and dairy products [45]. Parental education is needed so that they serve fruit and vegetables, not only at meals, but also at snacking occasions.

In this study, we reported common characteristics and differences in snacking patterns across countries and age groups. WHO recommendations focus on a healthy diet, promoting fresh fruits and raw vegetables as snacking foods instead of sugary and salty snacks [46]. Because intakes of calcium and some vitamins, particularly vitamin D, are low among children in the studied countries [44,47,48,49], snacking can be an opportunity to fill the nutritional gaps by prompting the right food choices. National guidelines on snacking need to be established based on the country-specific situation. For example in China, since over 95% of children have inadequate calcium intake and milk consumption is low [44], the national guidance is to promote milk and dairy consumption during snacking occasions along with fruits, vegetables, and nuts [10]. Hess and Slavin suggested that the combination of yogurt and fruit or vegetables for snacks would address the micronutrient intake inadequacy in US children [50]. Our findings support this suggestion to use the snacking occasion to improve the consumption of fruits, dairy, and other nutrient dense foods and provides insights for nutrition and public health professionals and authorities to develop guidelines and recommendations on snacking occasions that address the nutritional needs in children at different ages and in different countries.

This study has several strengths and limitations. Firstly, in order to provide an overview of snacking patterns in different countries, the USDA WWEIA food grouping system was used across all countries. However due to differences in each national food grouping method and local food culture, the risk of misclassification exists. Secondly, there is no universal definition of snacking in the scientific nutrition literature; it could be defined based on the time of day [51] or based on its contribution to total daily energy intake [52]. In this study, we applied a self-report based definition in all countries. The perception of snacking could be different among the respondents [53], but the risk for misclassification should be minimized by using trained interviewers during the dietary data collection in each national survey. Thirdly, to provide information on the general child population comparing countries, per capita data were used to describe the country snacking patterns. The individual food consumption and nutrient intake levels among snack consumers could show different scenarios.

## 5. Conclusions

To summarize, we found that snacking prevalence and the contribution of snacks to total daily energy and nutrients were different among children in the studied countries. Common characteristics could be seen in the foods that were commonly consumed in snacking occasions, but each country faced different issues. Reducing savory snack consumption and promoting fruit and vegetable consumption are needed across countries, especially among older children. In Australia, Mexico, and the US, limiting the consumption of sugary foods and beverages is needed, whereas in China snacks could be used to improve dairy consumption. To improve the quality of the foods and beverages consumed as snacks, clear snacking recommendations together with nutrition education that involves children, schools, parents, and caregivers, along with food industry engagement are needed.

## Figures and Tables

**Table 1:** Characteristics of the study populations and their snacking habits.

Characteristics	4–8 Year-Olds				9–13 Year-Olds			
	Australia	China	Mexico	US	Australia	China	Mexico	US
Total, *n*	789	769	2146	857	787	691	1718	829
Sex								
Boys (%)	52	52	49	52	51	52	51	52
Girls (%)	48	48	51	48	49	48	49	48
Snacking habits								
Consuming (%)	99.3	66.1	76.4	97.9	98.7	57.3	75.5	95.2
Energy from snacks (kcal) ^a^	565 ± 13	159 ± 8	276 ± 11	460 ± 15	717 ± 18	131 ± 8	328 ± 20	470 ± 20
TEI (%) ^b^	32.9 ± 0.6	11.9 ± 0.5	15.4 ± 0.5	26.6 ± 0.6	33.7 ± 0.6	7.9 ± 0.4	14.9 ± 0.8	24.0 ± 1.0

Weighted data are presented in this table except for China. ^a^ Energy (kcal) per capita per day, mean ± SE; ^b^ % Total energy intake (TEI) from snacks, mean ± SE.

**Table 2:** Nutrient intakes from snacking occasions among children (per capita) in Australia, China, Mexico, and the US.

Nutrient	Australia		China		Mexico		US	
	4–8 Years	9–13 Years	4–8 Years	9–13 Years	4–8 Years	9–13 Years	4–8 Years	9–13 Years
Total sugars (kcal)	203 ± 5	242 ± 6	56 ± 3	47 ± 3	96 ± 4	96 ± 6	162 ± 7	163± 8
Added sugars (kcal)	99± 4	134 ± 5	23 ± 2	15 ± 2	56 ± 3	58 ± 4	103 ± 6	124 ± 7
Total fat (kcal)	73 ± 2	100 ± 3	47 ± 3	36 ± 3	85 ± 5	112 ± 9	146 ± 6	157 ± 9
Saturated fat (kcal)	36 ± 1	47 ± 2	20 ± 1	13 ± 1	38 ± 2	48 ± 4	55 ± 4	58 ± 3
Protein (kcal)	51 ± 2	68 ± 2	18 ± 2	14 ± 2	23 ± 1	27 ± 2	38 ± 2	41 ± 3
Fiber (g)	6.3 ± 0.2	7.1 ± 0.2	1.5 ± 0.2	1.7 ± 0.2	2.8 ± 0.1	3.0 ± 0.2	3.1 ± 0.2	3.1 ± 0.2
Calcium (mg)	250 ± 9	302 ± 10	82 ± 8	56 ± 6	128 ± 6	117 ± 9	221 ± 16	223 ± 16
Sodium (mg)	495 ± 15	655 ± 23	150 ± 9	172 ± 12	255 ± 14	361 ± 30	438 ± 29	499 ± 25
Potassium (mg)	718 ± 19	850 ± 23	223 ± 22	232 ± 26	343 ± 15	362 ± 30	467 ± 24	412 ± 22
Zinc (mg)	1.7 ± 0.1	2.1 ± 0.1	0.8 ± 0.1	0.6 ± 0.1	1.1 ± 0.1	1.3 ± 0.1	1.5 ± 0.1	1.7 ± 0.1
Iron (mg)	2.1 ± 0.1	2.6 ± 0.1	1.6 ± 0.3	1.4 ± 0.3	1.7 ± 0.1	1.9 ± 0.2	2.6 ± 0.1	2.5 ± 0.2
Vitamin C (mg)	34.7 ± 1.7	39.3 ± 2.3	9.7 ± 0.9	14.1 ± 1.7	26.5 ± 1.9	31.2 ± 5.3	22.6 ± 2.0	19.9 ± 2.1
Vitamin D (µg)	N/A	N/A	0.4 ± 0.0	0.2 ± 0.0	6.8 ± 0.9	11.0 ± 5.5	1.0 ± 0.1	0.8 ± 0.1
Vitamin E as alpha-tocopherol (mg)	2.5 ± 0.1	3.0 ± 0.1	2.3 ± 0.2	2.0 ± 0.2	1.0 ± 0.1	1.5 ± 0.1	1.7 ± 0.1	2.0 ± 0.2

Data presented as mean ± SE; N/A: data not available.

**Table 3:** Top 10 of the most frequently reported foods and beverages consumed during snacking and their energy contributions among children in Australia, China, Mexico, and the US.

Country	Ranking	4–8 Year-Olds			9–13 Year-Olds		
		Food Group	Consuming of Each Food Group (%)	Energy (kcal) per Capita	Food Group	Consuming of Each Food Group (%)	Energy (kcal) per Capita
Australia	1	Water ^1^	82.8	0 ± 0	Water ^1^	83.2	1 ± 0
	2	Fruits	69.7	81 ± 3	Fruits	58.9 ***	75 ± 3
	3	Cookies & Brownies	42.8	69 ± 5	Cookies & Brownies	42.1	78 ± 5
	4	Savory Snacks	30.2	57 ± 5	Sweetened Beverages	36.2 **	44 ± 3
	5	Sweetened Beverages	29.9	32 ± 3	Candy	31.7	57 ± 5 ***
	6	Candy	27.8	33 ± 3	Milk ^2^	30.7	68 ± 5 ***
	7	Milk ^2^	26.6	49 ± 4	Savory Snacks	30.3	60 ± 5
	8	Ice Cream and Other Desserts	24.6	35 ± 3	Ice Cream and Other Desserts	25.2	47 ± 4
	9	Crackers	23.6	28 ± 3	Crackers	20.9	38 ± 4
	10	Breads	18.5	33 ± 4	Breads	19.5	47 ± 5
China	1	Fruits	42.9	30 ± 2	Fruits	41.3	36 ± 2
	2	Milk ^2^	16.0	24 ± 4	Milk ^2^	8.4 ***	11 ± 2
	3	Crackers	11.4	27 ± 3	Yogurt	5.9	7 ± 1
	4	Yogurt	5.5	6 ± 1	Crackers	3.4 ***	11 ± 3
	5	Cakes and Pies	4.5	11 ± 2	Ice Cream and Other Desserts	3.1	3 ± 1
	6	Breads	4.2	10 ± 2	Savory Snacks	2.3	7 ± 2
	7	Plant-based Protein Foods	3.9	6 ± 1	Cakes and Pies	2.3 *	5 ± 2
	8	Dairy Drinks and Substitutes	2.8	2 ± 1	Meats ^3^	2.0	3 ± 1
	9	Ice Cream and Other Desserts	2.8	3 ± 1	Candy	1.9	5 ± 2
	10	Candy	2.1	2 ± 1	Plant-Based Protein Foods	1.6 **	4 ± 2
Mexico	1	Water ^1^	37.0	0 ± 0	Water ^1^	36.6	0 ± 0
	2	Fruits	24.3	32 ± 3	Savory Snacks	24.2 ***	86 ± 10 ***
	3	Sweetened Beverages	19.9	28 ± 3	Sweetened Beverages	20.0	30 ± 3
	4	Candy	19.9	14 ± 2	Fruits	19.5 ***	33 ± 5
	5	Savory Snacks	18.5	45 ± 4	Candy	16.6 **	14 ± 2
	6	Milk ^2^	8.1	14 ± 2	Cookies & Brownies	6.0	27 ± 6
	7	Cookies & Brownies	7.5	26 ± 4	Cakes and Pies	5.9	22 ± 4
	8	Cakes and Pies	7.1	24 ± 3	Ice Cream and Other Desserts	5.8	9 ± 2
	9	Ice Cream and Other Desserts	6.2	10 ± 2	Pizza and Sandwiches	5.4	33 ± 13
	10	Pizza and Sandwiches	4.8	17 ± 3	Coffee and Tea	4.0	2 ± 1
US	1	Water ^1^	64.8	0 ± 0	Water ^1^	65.3	1 ± 0
	2	Candy	30.4	56 ± 16	Savory Snacks	31.0	58 ± 8
	3	Fruits	27.6	23 ± 1	Candy	29.3	45 ± 6
	4	Savory Snacks	27.6	56 ± 9	Sweetened Beverages	28.4	46 ± 6
	5	Cookies & Brownies	25.1	53 ± 8	Ice Cream and Other Desserts	20.1	46 ± 4
	6	Sweetened Beverages	24.8	25 ± 2	Fruits	18.1 ***	16 ± 2
	7	Milk ^2^	22.7	30 ± 3	Cookies & Brownies	17.3 ***	37 ± 6
	8	Ice Cream and Other Desserts	22.2	35 ± 3	Milk ^2^	12.3 ***	16 ± 2
	9	Crackers	17.8	24 ± 2	Cheese	12.2	12 ± 2
	10	100% Juice	15.1	17 ± 2	Crackers	10.5 ***	23 ± 6

^1^ Water: includes plain water, flavored or enhanced water. ^2^ Milk: includes milk and flavored milk. ^3^ Meats: includes meats, poultry, eggs, cured meats, and poultry. * *p* < 0.05, ** *p* < 0.01, *** *p* < 0.001 when comparing percent of consuming of the same food group between younger and older children of the same country.
